# Ethanol extract of basil (*Ocimum Basilicum* L.) leaves inhibits endometriosis growth in a mouse model by modulating vascular endothelial growth factor (VEGF) expression

**DOI:** 10.25122/jml-2023-0225

**Published:** 2023-08

**Authors:** Dewi Qurotul A’yuni, Ashon Sa’adi, Widjiati Widjiati

**Affiliations:** 1 Faculty of Medicine, Airlangga University, Surabaya, Indonesia; 2 Department of Obstetrics and Gynecology, Faculty of Medicine, Airlangga University, Surabaya, Indonesia; 3 Department of Veterinary Science, Faculty of Veterinary Medicine, Airlangga University, Surabaya, Indonesia

**Keywords:** basil leaf extract, reproductive health, VEGF, endometriosis lesion extent

## Abstract

The objective of this study was to examine the effect of administering an ethanol extract obtained from basil leaves on the expression of vascular endothelial growth factor (VEGF) and the severity of endometriosis lesions in a mouse model. A total of 28 female mice, aged 2-3 months and weighing 20-30 grams, were randomly divided into four groups: the control group (C), treatment group 1 (T1) receiving a dose of basil leaf ethanol extract (0.21 mg/g-BW), treatment group 2 (T2) receiving a higher dose (0.42 mg/g BW), and treatment group 3 (T3) receiving the highest dose (0.84 mg/g-BW). Each group underwent a 14-day treatment period, and tissue samples were collected on the 29^th^ day. An immunohistochemical examination was conducted to assess the expression of VEGF and evaluate the severity of endometriosis lesions. The statistical analysis of VEGF expression revealed a significant difference (p=0.026; p<0.05), with the most pronounced effects observed when administering basil leaf ethanol extract at doses of 0.21 mg/g-BW and 0.42 mg/g-BW. Although not statistically significant (p=0.271; p<0.05), a reduction in the severity of endometriosis lesions was observed following the administration of basil leaf ethanol extract at doses of 0.21 mg/g-BW and 0.42 mg/g-BW. Administering basil leaf ethanol extract at doses of 0.21 mg/g-BW and 0.42 mg/g-BW effectively decreased VEGF expression and limited the severity of endometriosis lesions.

## INTRODUCTION

Endometriosis, a prevalent gynecological disorder characterized by glands and stroma outside the uterine cavity [1], is associated with acute and chronic pelvic pain, infertility, dysmenorrhea, dyspareunia, abnormal vaginal bleeding, and in a severe stage, patients may experience gastrointestinal and urological symptoms [2]. The exact prevalence of endometriosis is not known; however, it is estimated that 6% -10% of reproductive-age women have endometriosis [3]. The recurrence rate of endometriosis is still relatively high, with 20.5% of patients reporting pain within three years and 43.5% within five years. Furthermore, 76% of patients have symptoms of recurrence associated with pain [4].

Angiogenesis has an important role in the development of endometriosis lesions and shows the occurrence of vascularization. Angiogenesis is primarily mediated by vascular endothelial growth factor (VEGF) and its receptor (VEGFR) [5, 6]. Ectopic endometrial cells can implant due to several factors, such as delayed menstruation, hormonal factors, immune system disorders, and chronic inflammation [1]. The inflammatory process and angiogenesis in endometriosis are played by activated macrophages by stimulating VEGF expression and inducing endothelial cell proliferation. These activated macrophages synthesize high concentrations of inducible nitric oxide synthase (iNOS), resulting in an increased production of nitric oxide (NO) and elevated levels of cyclooxygenase-2 (COX-2) and prostaglandin E2 (PGE2) [7]. The physiological function of VEGF is to induce angiogenesis and modulate newly formed blood vessels by controlling microvascular permeability, fibrin matrix formation, and endothelial cell proliferation [8]. Therefore, anti-VEGF therapy has the potential to slow the development of endometriosis and become a new strategy for endometriosis treatment [9-11].

Basil (*Ocimum basilicum* L) leaves are plants that contain high levels of flavonoids. Basil leaf flavonoids such as nevadensin, salvigenin, cirsileol, eupatorin, kaemferol, rutin, cirsimaritin, quercetin, and glycosides, are one of the main secondary metabolites identified in basil [12, 13]. Quercetin is one of the many flavonoids found in vegetables and fruits. Quercetin has been shown to inhibit angiogenesis by targeting the VEGF-R2-regulated AKT/mTOR/P70S6K signaling pathway [14, 15].

Basil leaves have been shown to be useful for their antiproliferative, anti-inflammatory, anti-angiogenic, antimicrobial, antioxidant, and immunomodulatory activities [16]. The antiangiogenic properties of basil flavonoids act through several key mechanisms. Firstly, they inhibit cyclooxygenase-2 (COX) [17]. Secondly, they influence nuclear factor-kappa B (NF-KB) signaling [18, 19]. Thirdly, they suppress VEGFR2 phosphorylation, inhibit VEGFR2 mRNA expression, and inhibit phosphorylation of downstream kinases AKT, extracellular signal-regulated kinase (ERK), mammalian target of rapamycin (mTOR) [19]. Other studies show flavonoids have anti-inflammatory effects by inhibiting lipoxygenase (LOX), COX-2, and NF-kB [20, 21]. Research conducted by Zhang *et al*. demonstrated that flavonoids (quercetin) can induce apoptosis of colon cancer cells in humans through NF-kB inhibition [22].

Given that patients with endometriosis commonly experience inflammatory and angiogenesis disorders, it is reasonable to consider utilizing basil leaf herbal therapy, which contains flavonoids known for their anti-inflammatory and anti-angiogenic properties, as a complementary alternative medicine. This approach offers several advantages, including its natural origin, comfort, affordability, and a reduced likelihood of side effects, such as interference with ovulation [11, 23, 24]. While flavonoids are known to improve the profile of various diseases such as autoimmune, inflammatory, and angiogenesis-related conditions, the specific effects of flavonoids contained in basil leaves on VEGF expression in endometriosis have not been studied [7, 14, 25-28]. This study aimed to investigate the effect of graded doses of basil leaf extract on VEGF expression and the extent of endometriosis lesions in a mouse model.

## MATERIALS AND METHODS

### Study design and location

This research followed an experimental design with a post-test-only control group. The study was conducted at the Experimental Animal Cage and Veterinary Pathology Laboratory, Faculty of Veterinary Medicine, Airlangga University, Surabaya.

### Animal models and research flow

**Figure 1:**
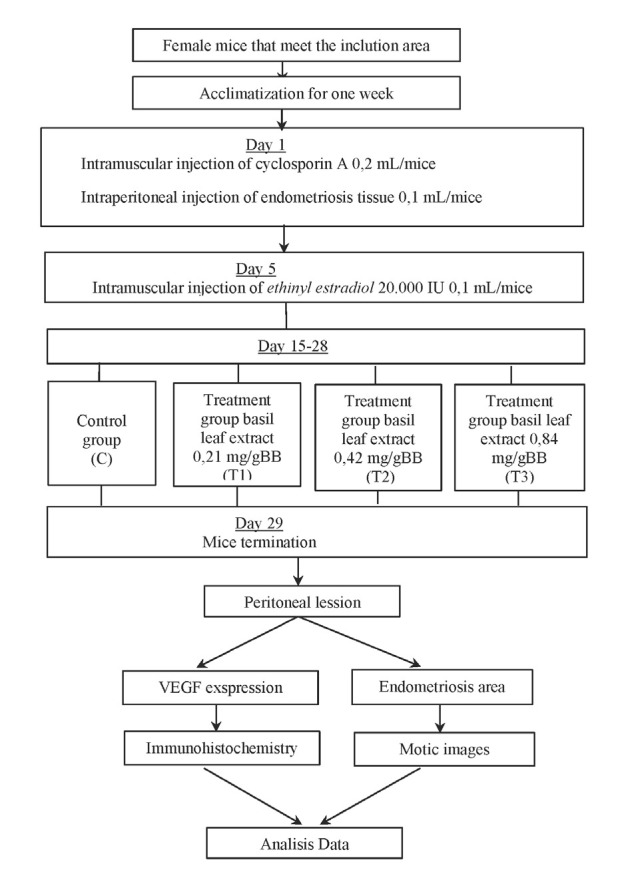
Research flowchart

The samples included in this study were 28 mice (Mus musculus) aged approximately 2-3 months and weighing 20-30 grams. The mice were housed in a controlled cage with a stable environment, adequate ventilation, and room temperatures of 22oC-25oC. Throughout the study, the mice had access to food and water ad libitum [29]. Mice were divided into 4 groups: the control group (C) and three treatment groups (T1, T2, and T3) receiving ethanol extract of basil leaves at varying doses (0.21 mg/g-BW, 0.42 mg/g-BW and 0.84 mg/g-BW). Treatment started on the 15^th^ day of the research and lasted for 2 weeks. Euthanasia was performed via cervical dislocation following anesthesia using ether/chloroform in a closed container until the mice ceased to move. The research procedures and stages conducted in the research are shown in Figure 1.

### Preparation of endometriosis mouse model

Endometriosis mouse models were made after acclimatization was reached. Each mouse received an intramuscular injection of Cyclosporine A (0.2 mL) and an intraperitoneal injection of endometrial tissue (0.1 mL) on day 1. Subsequent injections with ethinyl estradiol 20,000 IU 0.1 mL were administered on days 1 and 5 for each mouse [30]. After these injections, the mice were monitored until the 14^th^ day with regular feeding and access to water.

### Preparation of ethanol extract of basil (*Ocimum Basilicum* L) leaves

Basil leaves were obtained from plantations in Binjai, North Sumatra. The leaves were processed using the maceration method with 96% ethanol solvent at the Pharmaco Laboratory of Airlangga University, Surabaya. The extraction process started by drying basil leaves for 2 days at 30-35^o^C, followed by crushing with a blender and sieving. The dry sample was put into a 1-liter Erlenmeyer glass soaked in 96% ethanol and left for the night until it settled. The top layer of ethanol was removed and mixed with the active ingredients, and this soaking process was carried out up to 3 times. Next, ethanol was removed with a vacuum evaporator at a temperature of 90^o^C or according to the boiling point of the solvent. When the ethanol solution stopped dripping on a flask, a thick extract was obtained and then stored. Doses for the basil leaf extract were determined based on a previous study by Eftekhar *et al*. (2019), which demonstrated anti-inflammatory effects in rats at doses of 150, 300, and 600 mg/kg/BW [20]. These doses were converted from rats (200 grams) to mice (20 grams) to achieve the doses of 0.21 mg/g/BW, 0.42 mg/g/BW, and 0.84 mg/g/BW.

### Immunohistochemical examination of VEGF expression

Immunohistochemical examination of VEGF expression was carried out on the 15^th^ day after treatment. The slides were washed with phosphate-buffered saline (PBS) pH 7.4 twice for 5 minutes and then dripped with endogenous peroxidase methanol H2O2 0.3% for 15 minutes. After that, they were rinsed with running water for 5 minutes and rewashed with distilled water. VEGF monoclonal antibody was dropped on the slides, which were incubated at 4^o^C for 18 hours. The slides were washed with PBS and dripped with secondary antibody (labeled with biotin) and streptavidin for 10 minutes, and then they were washed with PBS. After that, Diethyl Amino Benzyn peroxidase enzyme substrate was given for 15 minutes, and then they were washed with water. Hematoxylin was dropped on them, and they were washed with running water. Furthermore, mounting was carried out using Entellan and covered with a cover glass [31]. Finally, analyses were conducted using a light microscope Nikon Eclipse E-100, Optilab viewer 2.3 with 400x magnification.

### Evaluation of immunohistochemical staining

Staining intensity was calculated using the Remmele scale index (Immuno Reactive Score/IRS) calculated by multiplying the percentage score of positive cells with the color reaction intensity score. Positive cell percentage score: Score 0: No positive cells; Score 1: Positive cells <10%; Score 2: Positive cells between 11% and 50%; Score 3: Positive cells between 51% and 80%; and Score 4: More than 80% positive cells. The color reaction intensity score ranged from Score 0: no color reaction, Score 1: Low color intensity, Score 2: Moderate color intensity, and Score 3: Strong color intensity [32, 33]. VEGF expression, appearing as brownish-red staining in endometriosis lesions, was assessed in ten fields of view. The results were documented using a DSLR camera.

### Motic images on endometriosis lesion extent

Endometriosis lesions were obtained by performing abdominal surgery on mice and opening the peritoneum. Endometriosis lesions were measured after the tissue was washed with PBS, placed on millimeter block paper, and photographed. Data were processed using the Motic Images program.

### Statistical analysis

All data obtained were analyzed using SPSS 25.0 software. VEGF expression was assessed using the one-way analysis of variance (ANOVA) test, followed by the least significant difference (LSD) post hoc test for identifying significant differences among groups. The Kruskall-Wallis test was used to evaluate the extent of endometriosis lesions. A significance level of p<0.05 was considered statistically significant.

## RESULTS

### VEGF expression

**Figure 2:**
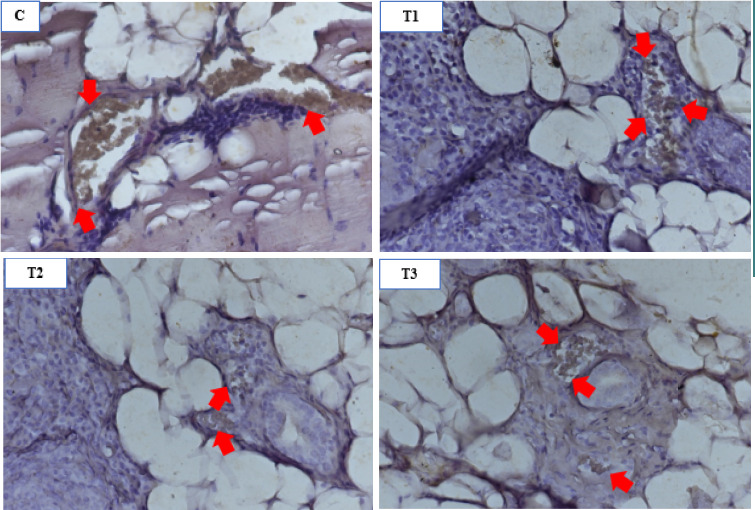
Differences in VEGF expression. The control group (C) shows very strong VEGF expression in the endothelial tunica of blood vessels in the endometriosis tissue marked with brown chromogen (arrows), (T1) shows less VEGF expression compared to the control group, (T2) shows weak VEGF expression, and (T3) shows strong VEGF expression; IHC 400x

The immunohistochemical staining results of the peritoneal tissue (Figure 2) revealed VEGF expression within the vascular endothelium of the endometriosis tissue. The mean VEGF expression in the control group (C) was higher (3.04±1.68) than that of T1 (1.41±0.31), T2 (1.58±0.44), and T3 groups (2.10±0.97) (Table 1). The one-way ANOVA test showed a significant difference between the control and treatment groups (p=0.026). The LSD post hoc test showed a significant difference between the 0.21 mg/g-BW treatment group (T1) (p=0.006) and the 0.42 mg/g-BW treatment group (T2) (p=0.013) compared to the control group. The 0.84 mg/g-BW treatment group (T3) showed the highest VEGF expression, although it was still below the VEGF expression of the control group.

**Table 1:** VEGF expression and endometriosis lesion extent in endometriosis mouse model

	Groups			
	C (n=7)	T1 (n=7)	T2 (n=7)	T3 (n=7)
VEGF expression	3.04±1.68	1.41±0.31*	1.58±0.44*	2.10±0.97
Endometriosis Lesion Extent	5.41±4.87	1.13±1.97	2.14±3.96	6.63±8.76

Note: Significant differences with the control group were marked with (*) (p<0.05). (C): control group; (T1): treatment group received ethanol extract of basil leaves at a dose of 1 0.21 mg/gBW; (T2): treatment group received ethanol extract of basil leaves at a dose of 0.42 mg/gBW; (T3): treatment group received ethanol extract of basil leaves at a dose of 1 0.84 mg/gBW

### Endometriosis lesion extent

**Figure 3:**
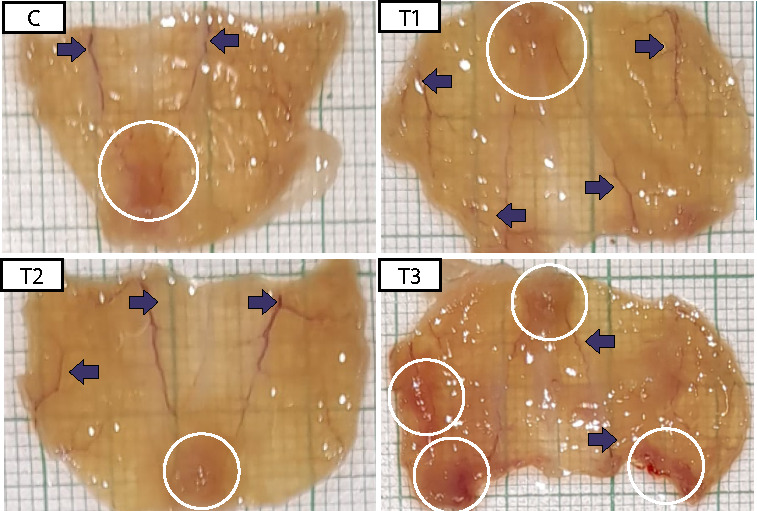
The difference in endometriosis lesion extent decreased significantly in T1 (3.34 mm^2^) and T2 (4.91 mm^2^) compared to C (14.01 mm^2^). The endometriosis lesion extent increased significantly with dosage, as evidenced by T3 (20.5 mm^2^). Arrows indicate hypervascularization.

The extent of endometriosis lesions in the peritoneal tissue is presented in Figure 3. The mean endometriosis lesion extent in the control group (C) (5.41±4.87) was higher than in T1 (1.13±1.97) and T2 (2.14±3.96) (Table 1). The Kruskal-Wallis test showed no significant differences between the control and treatment groups (p=0.271). However, the mean results suggested that the treatment group receiving 0.21 mg/g-BW (T1) and the group receiving 0.42 mg/g-BW (T2) exhibited lower means compared to the control group (C), whereas there was an increased mean in the treatment group (T3) administered with 0.84 mg/g-BW (T3).

## DISCUSSION

The treatment of endometriosis is currently symptomatic, and subsequently, the progress of endometriosis itself must be suppressed in the long term to reduce the recurrence rate [34]. Given the role of VEGF in the pathogenesis of endometriosis, its inhibition to suppress vascular development may be a new therapeutic strategy for treating endometriosis [6]. To our knowledge, this study is the first to show the effect of an ethanol extract derived from basil leaves on VEGF expression in endometriosis. In this study, the highest VEGF expression was found in endometriosis mouse groups not treated with ethanol extract of basil leaves, supporting other studies showing increased VEGF expression in the peritoneum of patients with endometriosis [5, 8, 35]. VEGF is the main stimulus for angiogenesis and increased vascular permeability in endometriosis and is strongly expressed by endometriotic lesions and activated macrophages. Activated macrophages play an important role in the secretion of proinflammatory and proangiogenic cytokines [5, 6]. Consistent with a study by Lin *et al*., which used an endometriosis mouse model, it was reported that tumor necrosis factor alpha (TNF-α) and interleukin-6 (IL-6) were secreted by macrophages and resulted in VEGF upregulation of neutrophil and macrophage infiltration [36].

In this study, the administration of ethanol extract derived from basil leaves resulted in lower VEGF expression compared to the control group. There were significant differences in VEGF expression between the endometriosis mouse groups treated with doses of 0.21 mg/g-BW and 0.42 mg/g-BW compared to the control group. The activity of basil leaf flavonoids has antiangiogenic effectiveness to reduce VEGF expression through several inhibiting mechanisms, such as inhibition of COX-2, NF-KB, VEGFR2 phosphorylation, VEGFR2 mRNA expression, and phosphorylation of downstream kinases AKT, ERK, mTOR [17, 19].

This is in line with research by Nangia-Makker *et al*., which stated that the ethanol extract of *Ocimum sanctum*, which belongs to the same genus as *Ocimum basilicum*, can inhibit tumor growth and angiogenesis by affecting tumor cell proliferation, migration, morphogenesis, stromal apoptosis and induction of COX-2 [25]. This is also in accordance with Gong *et al*., which stated that Scutellariae Radix (SR) is the dried root of *Scutellaria baicalensis* Georgi, a traditional Chinese medicine containing flavonoids. Scutellariae Radix could be a promising target in developing drugs to reduce angiogenesis-induced VEGF levels triggered by chronic inflammation by suppressing the expression of COX-2, iNOS, HIF-1α, and VEGF [26]. Research by Pratheeshkumar *et al*. stated that flavonoids (quercetin) can inhibit tumor growth and angiogenesis by targeting the AKT/mTOR/P70S6K signaling pathway regulated by VEGF-R2 in a mouse model of prostate tumor xenograft [14]. Furthermore, research by Ilhan *et al*. on the yellow sweet clover plant (*Melilotus officinalis* L), which has flavonoid activity, could reduce levels of TNF-α, VEGF, and IL-6 in endometriosis rat models [27].

The endometriosis model in this study was a mild endometriosis model. The endometriosis lesions, which appeared opaque red and well-established, exhibited characteristics consistent with the infiltration of immune cells, increased microvessel density, enhanced vascularization, elevated cytokine production, higher cell proliferation index, increased prostaglandin levels, and the formation of angiogenesis. These observations align with the criteria outlined in the classification of endometriosis by the American Society of Reproductive Medicine (ASRM) [37]. In this study, the extent of endometriosis lesions did not exhibit any significant differences between the control and treatment groups that received the basil leaf ethanol extract. However, the results did show that the mean extent of endometriosis lesions in the treatment group at dose 1 (0.21 mg/gBW) and the treatment group at dose 2 (0.42 mg/gBW) was lower when compared to the control group. This reduction in endometriosis lesion extent corresponds with the observed decrease in VEGF expression in the treatment groups administered with doses 1 and 2, which were the most influential doses.

The balance of pro-antiangiogenic factors and proinflammatory cytokines can determine whether endometriosis lesions develop and grow. Research conducted by Aminian *et al*. on asthma, involving the administration of ethanol extract derived from basil leaves, showed improvements in the pathological changes related to inflammation and lung immunity in rat models of asthma by suppressing signaling from the NF-kB pathway [28]. Flavonoids can provide anti-inflammatory activity through the mechanism of NF-κB inhibition. NF-κB is an important therapeutic target in chronic inflammatory diseases, which allows significant downregulation of pro-inflammatory cytokines produced by macrophages [38].

The results showed that the greatest endometriosis lesion extent was found in the treatment group administered basil leaf ethanol extract of 0.84 mg/g-BW or dose 3. Likewise, in terms of VEGF expression, the treatment group administered basil leaf ethanol extract of 0.84 mg/g-BW or dose 3 had the highest value compared to the other treatment groups, and it was considered a dose that was statistically insignificant. The increased VEGF expression and endometriosis lesion extent indicated that the angiogenesis process continued in the endometriosis mouse models administered with ethanol extract of basil leaves. This is because the VEGF production by macrophages can be regulated by several pathways. Similarly, the peritoneal environment in the presence of endometriosis can change significantly, which can lead to excessive activation of various kinase signaling pathways in endometriosis tissue [39]. The formation of extensive endometriotic lesions requires angiogenic factors such as VEGF, cytokines, and metalloproteinases. The interrelationship of several of these factors allows capillary growth from existing blood vessels for development into ectopic implants [40].

In macrophages, the production of VEGF can involve multiple pathways. One such pathway is through the activation of nuclear factor-κB (NF-κB). This activation can be triggered by various factors, including lipopolysaccharide (LPS) and the interaction between CD40 ligands on the surface of macrophages, along with signaling via toll-like receptor 4 (TLR4) [38]. These factors collectively lead to the activation of NF-κB, which in turn contributes to the production of VEGF. Another pathway regulating VEGF expression is mitogen-activated protein kinases (MAPKs). MAPKs induce localized transcription factors in the nucleus by activating the expression of specific target genes to be modulated. MAPK signaling pathways can play different roles in immune-mediated inflammatory responses, cell proliferation, cell differentiation, angiogenesis, and apoptosis, which are the cellular events associated with endometriosis [41]. This approach is supported by Leconte *et al*., who included human ectopic endometrial cell cultures and mouse models and demonstrated that the MAPK/ERK signaling pathway can be activated through VEGFR and causes the growth of endometrial lesions by influencing angiogenesis [42]. The high VEGF expression and endometriosis lesion extent in this study were due to the inhibition of VEGF production in one pathway, namely NF-kB, which was inhibited by flavonoids in the ethanol extract of basil leaves, while in the MAPK pathway, there was no inhibition of VEGF production; thus there was an increase.

The high VEGF expression and endometriosis lesion extent observed in treatment group 3 (T3) can be attributed to the phytoestrogens contained in basil leaves, even though these may not be clear enough at lower doses. VEGF expression and endometriosis lesion extent were the lowest in the treatment group receiving dose 1 (0.21 mg/g-BW). Research by Osei Akoto *et al*. states that steroids and triterpenoids are some of the phytochemical contents of basil leaves. These steroids (sitosterol) can stimulate the production of estrogens through the aromatase process and can exacerbate endometriosis [43]. Endometriosis is a disease characterized by elevated estrogen levels, leading to increased endometrial tissue growth and the formation of ectopic lesions [44]. Estrogen directly modulates angiogenesis through its effect on endothelial cells by inducing endothelial proliferation and migration mediated by estrogen receptors [45]. Andrianto *et al*. showed that high concentrations of estrogen and prostaglandins in experimental rats treated with ethanol extract of basil (*Ocimum basilicum* L) leaves had greater uterine vascularization by increasing VEGF expression [29]. Research by Machado *et al*. stated that severe, very active red endometriosis lesions contained the highest concentration of VEGF compared to mild endometriosis [5]. Furthermore, Machado *et al*. elevated VEGF levels can activate macrophages, binding to VEGFR-2 and regulating MMP-9 transcription. This induces extracellular matrix remodeling, promoting increased blood vessel density and the expansion of endometriosis lesions [7]. This is in accordance with the results obtained in treatment group 3 (T3) on the endometriosis lesion extent, which showed a high rate of endometriosis lesion extent and elevated VEGF expression.

This study is the first to show the effect of ethanol extract of basil leaves on VEGF expression in endometriosis. The findings of this study are significant because they can increase our understanding of the mechanism of ethanol extract of basil leaves in endometriosis and provide information on endometriosis. However, the use of high doses should be cautious. This study has several limitations. For example, we did not evaluate estrogen levels in endometriosis mice before and after being treated with basil leaf ethanol extract. Moreover, no patent dose related to basil leaf complementary therapy for endometriosis existed.

## CONCLUSION

The administration of ethanol extract derived from basil leaves at doses of 0.21 mg/g-BW and 0.42 mg/g-BW led to a significant reduction in VEGF expression, although it did not significantly affect endometriosis lesion extent. However, there was a decrease in endometriosis lesion extent at doses of 0.21 mg/g-BW and 0.42 mg/g-BW. At the highest dose, which is 0.84 mg/g-BW, it is necessary to be cautious as there was an increase in VEGF expression and endometriosis lesion extent. Thus, the researchers concluded that the administration of ethanol extract from basil leaves could inhibit the development of endometriosis lesions by modulating VEGF expression in endometriosis mouse models, and it is necessary to be wary of the use of high doses. It is necessary to conduct further research on the benefits of phytoestrogens present in basil leaf extract.

## Personal thanks

The authors would like to acknowledge the excellent teaching staff of Reproductive Health, Faculty of Medicine, Airlangga University, Obstetrics and Gynecology of Dr. Soetomo, and laboratory staff of the Veterinary Faculty of Ailangga University.
